# The relative effectiveness of psychotherapeutic techniques and delivery modalities for chronic pain: a protocol for a systematic review and network meta-analysis.

**DOI:** 10.12688/hrbopenres.12953.2

**Published:** 2020-08-17

**Authors:** Stephanie Haugh, Laura O'Connor, Brian Slattery, Michelle Hanlon, Jack Flynn, Sarah Quinn, Caroline Jennings, Brian E. McGuire

**Affiliations:** 1 Centre for Pain Research, School of Psychology, National University of Ireland, Galway, Galway, Ireland; 2 School of Psychology, Dublin City University, Dublin, Ireland

**Keywords:** Psychotherapy, Chronic Pain, eHealth, Systematic Review, Network Meta-Analysis

## Abstract

**Introduction**: There is increasing evidence for the use of psychotherapies, including cognitive behavioural therapy, acceptance and commitment therapy, and mindfulness based stress reduction therapy, as an approach to management of chronic pain. Similarly, online psychotherapeutic interventions have been shown to be efficacious, and to arguably overcome practical barriers associated with traditional face-to-face treatment for chronic pain. This is a protocol for a systematic review and network meta-analysis aiming to evaluate and rank psychotherapies (delivered in person and online) for chronic pain patients.

**Methods/ design:** Four databases, namely the Cochrane Central Register of Controlled Trials (CENTRAL), MEDLINE, EMBASE and PsycINFO will be searched from inception. Randomised controlled trials that have evaluated psychological interventions for pain management delivered online or in person will be included in the review. Data will be independently extracted in duplicate and the Cochrane Collaboration Risk of Bias Tool will be used to assess study quality. Measures of pain interference will be extracted as the primary outcome and measures of psychological distress will be extracted as the secondary outcome. A network meta-analysis will generate indirect comparisons of psychotherapies across treatment trials. Rankings of psychotherapies for chronic pain will be made available.

**Discussion: **A variety of psychotherapies, delivered both online and in person, have been used in an attempt to help manage chronic pain. Although occasional head to head trials have been conducted, little evidence exists to help identify which psychotherapy is most effective in reducing pain interference. The current review will address this gap in the literature and compare the psychotherapies used for internet delivered and in person interventions for chronic pain in relation to the reduction of pain interference and psychological distress. Results will provide a guide for clinicians when determining treatment course and will inform future research into psychotherapies for chronic pain.

**PROSPERO registration**: CRD42016048518 01/11/16

## Abbreviations

*RCT:* Randomised Control Trial

*NMA:* Network Meta-Analysis

*PRISMA:* Preferred Reporting Items for Systematic Reviews and Meta-Analyses

*PROSPERO:* Prospective Register of Systematic Reviews

*SMD:* Standardised Mean Difference

## Introduction

### Chronic pain

Chronic pain refers to pain (“an unpleasant sensory and emotional experience associated with actual or potential tissue damage, or described in terms of such damage” [ IASP, 2012; Merskey *et al.*, 1979]) that persists for more than three months. Chronic pain is highly prevalent, and one of the leading causes of long-term disability and reduced quality of life globally ( Blyth *et al.*, 2019; Hurwitz *et al.*, 2018; Raftery *et al.*, 2011). Chronic pain is considered a highly multifaceted disorder with multiple physical, emotional, behavioural and cognitive determinants ( Lumley *et al.*, 2011). Although pharmacological treatments can reduce pain severity, they do not yield concurrent improvements in reducing disability and emotional distress; psychological therapies are typically implemented in conjunction with medical treatments to yield the highest improvements in physical and emotional wellbeing ( Turk *et al.*, 2008).

### Psychotherapies for chronic pain

Psychological therapies for chronic pain typically focus on addressing the cognitive, behavioural, emotional and social factors thought to sustain physical disability ( Sturgeon, 2014). Cognitive behavioural therapy (CBT) is generally accepted as a treatment for chronic pain and is currently considered the “gold standard” in psychological interventions for pain ( Day *et al.*, 2012). Research has contributed to the emergence of new psychotherapeutic approaches for chronic pain management including Acceptance and Commitment Therapy (ACT; Wetherell *et al.*, 2011; Wicksell *et al.*, 2013), Behaviour Therapy (BT; Williams *et al.*, 2010), Mindfulness ( Henriksson *et al.*, 2016; Morone *et al.*, 2008) and Hypnosis ( Picard *et al.*, 2013).

Psychological therapies have been used to target a variety of aspects of chronic pain, including, pain interference, psychological distress, health related quality of life and pain catastrophizing. Although measures of pain intensity are often assessed, reducing pain intensity is typically of secondary importance in psychotherapeutic interventions ( Sturgeon, 2014). Recent systematic reviews in this area have returned promising results for the use of psychotherapies; for example Veehof *et al.* (2011) concluded that acceptance based interventions, including mindfulness based stress reduction (MBSR) and ACT, returned statistically significant effects in relation to pain related outcomes in comparison to pooled controls. Additional systematic reviews and meta-analyses have indicated that psychotherapeutic interventions have a positive effect on pain related outcomes ( Chiesa & Serretti, 2011; Hoffman *et al.*, 2007; Williams *et al.*, 2012).

### Psychotherapies online

The use of information technologies for the promotion and maintenance of health, and the prevention and management of disease is referred to as eHealth ( Catwell & Sheikh, 2009; Showell & Nøhr, 2012). As the use and advancement of digital technologies continues to grow, so too does the use of eHealth interventions to manage chronic conditions ( Srivastava *et al.*, 2015). The use of eHealth interventions enable patients to overcome a number of treatment barriers, including the cost of face-to-face treatment, unavailability of qualified clinicians, and travel and mobility issues ( Heapy *et al.*, 2015; Liaw & Humphreys, 2006; Stroetmann *et al.*, 2006). In terms of their effectiveness for improving chronic pain, a recent meta-analysis conducted by Eccleston *et al.* (2014) found that psychotherapeutic interventions implemented online yielded improved pain symptoms at post-treatment, and disability at post treatment and follow-up. A significant number of psychotherapies have been adapted into online formats, including iCBT ( Mourad *et al.* (2016); Vallejo *et al.* (2015), iACT ( Buhrman *et al.* (2013), iBT ( Williams *et al.* (2010)), and iMindfulness ( Henriksson *et al.* (2016)), these online interventions for chronic pain have been shown to be efficacious and have potential for use in a health care system.

### Why is it important to do this review?

Psychotherapies are generally accepted as a treatment option for chronic pain patients. CBT has historically been considered the gold standard treatment, however, with research into different psychotherapies yielding positive results, it is difficult to know the most appropriate direction in either a clinical or research setting. There has also been an increase in internet delivered interventions for chronic pain, however, there is a dearth of research evaluating the efficacy of online psychotherapies in comparison to their counterparts delivered in person. The current review will investigate the current available evidence using a network-meta analysis (NMA). In the context of a systematic review, NMA is a statistical technique enabling comparisons of multiple treatments through direct and indirect comparisons across trials that use a common comparator (for further discussion, see Jansen *et al.*, 2014; Naci *et al.*, 2013). This will facilitate comparisons regarding the relative efficacy of different psychotherapies and delivery modalities that would otherwise not be possible until further primary research is carried out.

### Objective

The objective of this study is to evaluate the relative effectiveness of existing online and in-person psychotherapeutic interventions for chronic pain management.

This will be done by reviewing the current available evidence from relevant papers and using the NMA method to create a network of direct and indirect comparisons, thereby creating a tentative ranking of interventions.

## Methods

### Protocol and registration

This systematic review and NMA will be conducted and reported in accordance with the Preferred Reporting Items for Systematic Reviews and Meta-Analyses (PRISMA) guidelines and the PRISMA Network Meta-Analysis extension statement (see Reporting guidelines; Hutton *et al.*, 2015; Moher *et al.*, 2009). In accordance with these recommendations, this review will use PICO to frame and report eligibility criteria; as such, participants, interventions, comparisons, outcome(s) and study design of included studies will be reported. The protocol for this study was registered with the International Prospective Register of Systematic Reviews (PROSPERO) database (registration number: CRD42016048518) on 1 November 2016.

### Types of studies

Only randomised controlled trials (RCTs) comparing a psychological intervention, with at least one of an alternative psychological intervention, waitlist control (WLC), treatment-as-usual (TAU) or non-psychological intervention (for example, exercise, education and medical therapy) will be eligible for inclusion. Eligible psychological interventions must have identifiable psychotherapeutic content such as behavioural (for example, biofeedback, relaxation or behaviour monitoring) or cognitive behavioural (for example, coping skills, cognitive re-constructing or problem solving) components. The interventions must be delivered in person or via the internet. There are no restrictions placed on treatment intensity, duration or number of psychotherapeutic techniques employed. Studies must be full-text journal articles available in English, published in peer-reviewed journals, and available using database access or through contacting study authors.

### Types of participants

Eligible participants must be adults (>18 years) living with chronic pain, as defined by the International Association for the Study of Pain ( IASP 2012; Merskey *et al.*, 1979). Studies which examine samples where chronic pain is induced by debilitating diseases such as cancer or multiple sclerosis will not be included due to differences in disease prognosis compared to other forms of chronic pain ( Treede *et al.*, 2015). As chronic headaches and migraine treatment is generally considered to be different to other chronic pain treatments ( Williams *et al.*, 2012), studies examining samples where headache is the primary disorder will not be included.

### Types of outcome measures

***Primary outcomes.*** Studies will only be included in the NMA if they contain a self-reported measure of pain related interference or similar, for example pain related disability or impact.

***Secondary outcomes.*** A second network will be created to investigate the secondary outcome which will include self-reported scales of psychological distress (including depression, anxiety, negative affect or psychological stress).

### Electronic searches

The following databases will be searched from inception: EMBASE, MEDLINE, CENTRAL (Cochrane Library), and PsycINFO. Searches strategies will be the same for all databases; however necessary changes will be made to account for differences in interfaces. The search strategy is described in Table 1 (see extended data ( O’Connor, 2019)).

**Table 1.** Search Strategy.

**Search Strategy**
Psychotherapy.sh OR (ACT.mp OR (acceptance and commitment.ab)) OR (CBT.mp OR cognitive*.ab) OR mindfulness.ab OR supportive.ab OR (DBT.mp OR dialectical.ab) OR behavio#r*.ab OR existential.ab OR humanistic.ab OR gestalt.ab OR psychoanalytic.ab OR therapy.mp
Pain/ OR pain measurement/ OR fibromyalgia/ OR (pain intensity.ab OR pain severity.ab OR pain outcome*.ab OR pain interference.ab OR physical functioning.ab) OR self-reported pain.ab OR chronic pain.mp
Randomized controlled trial.pt OR randomised controlled trial.pt OR controlled clinical trial.pt OR randomized.ab OR randomised.ab OR placebo.ab OR randomly.ab OR trial.ab OR groups.ab OR RCT.mp

### Searching other resources

Reference lists of relevant systematic reviews will be searched to identify additional relevant studies. The metaRegister of Controlled Trials (mRCT), clinicaltrials.gov and the WHO International Clinical Trials Registry Platform (ICTRP) will also be searched. This review will only include studies published in peer-reviewed journals; dissertations, unpublished papers and on-going studies will be excluded.

### Study selection

Search results will be imported into Endnote X7 to create a library of all studies found, and individual folders created to track included and excluded studies. The research team will screen titles and abstracts, first to identify duplicate studies and then screen for any studies that do not satisfy the inclusion criteria, with 10% of studies screened in duplicate to ensure consistency. Studies not available in English will be excluded at this time. Two authors (SQ and CJ) will independently screen full papers of the remaining studies for inclusion in agreement with the inclusion criteria. Studies will be sequentially excluded via the exclusion categories if they do not satisfy the criteria. Disagreements between screeners will be discussed, and where a decision cannot be reached, a third reviewer (SH) will mediate. A flow chart will graphically depict the exclusion of studies.

### Data collection process

Data will be extracted from the included studies in duplicate into a pre-prepared data extraction Excel sheet, to be piloted with three studies and amended if necessary before data extraction begins proper. Authors of included papers will be contacted if it is necessary to recover missing data.

### Data items

Data will be extracted in accordance with the following categories:

- Participants: sample size, percentage female, mean age, diagnosis, mean years of pain.
- The type of psychotherapy/control employed in each arm.
- Primary measure used to record each outcome.
- Means and standard deviations at post intervention of suitable outcome measures and the measure used to collect the data.

### Geometry of the network

A network diagram will visually represent available evidence, depicting the possible comparisons of any two psychotherapies (for example, CBT vs ACT) and the comparison of delivery methods of psychotherapy (for example, CBT vs iCBT). Any arms not directly connected to the network will not be included in analysis.

### Risk of bias in included Studies

As participants and intervention providers are unable to be blinded in studies assessing psychological interventions, the bias domain assessing blinding of participants/personnel will be omitted from this review. Similarly, as this review focuses solely on self-report measures, the bias domain assessing blinding of outcome assessors will also be omitted. Risk of bias will be assessed by two independent reviewers. Two authors (JF & BO’G)will independently use the Cochrane Collaboration Risk of Bias tool to classify studies as being of low, high or an unclear risk of bias based on the following six domains; random sequence generation, allocation concealment, incomplete outcome data, reporting bias, use of intention-to-treat analysis, uneven distribution of potential cofounders at baseline. Where disagreements regarding the risk of bias are encountered, a third author (SH) will mediate. For more detail see Slattery *et al.* (2017).

### Quality of evidence

The Grading of Recommendations Assessment, Development and Evaluation (GRADE) criteria will be used to judge the quality of evidence for the direct, indirect and NMA effect estimates yielded within the network ( Puhan *et al.*, 2014). The quality of the direct effect estimates will be judged based on the methodological limitations of the included trials, imprecision, indirectness, inconsistency, and publication bias. Providing the certainty of the direct estimates is high, and the contribution of the direct estimates is at least equal to that of the indirect estimates, the quality of evidence for the network estimates will be based solely on the direct evidence ( Brignardello-Petersen *et al.*, 2018). Otherwise, the quality of the indirect evidence will be determined using the direct comparisons with the highest contribution in generating those comparisons, and the quality of the network estimates will be determined based on the quality of the direct and indirect comparisons making the largest contribution. In this instance, imprecision will not be considered when evaluating the quality of the network estimates ( Brignardello-Petersen *et al.*, 2018). Additionally, where incoherence between direct and indirect estimates is evident, the quality of the estimate making the highest contribution will be used to inform the quality of the network estimate but will be downgraded regardless ( Brignardello-Petersen *et al.*, 2018). In the instance of sparse networks and incoherence resulting in widened network estimate confidence intervals, exploratory sensitivity analyses based on the removal of trials with common covariance will be considered ( Brignardello-Petersen *et al.*, 2019).

### Summary measures

This review will use Stata 13 for all analyses. Mean differences between groups and 95% confidence intervals will be reported. Where outcomes are obtained using a variety of measures, standardised mean differences (SMD) will be calculated and reported with their 95% confidence intervals. To adjust for the SMD direction, the mean score of measures in which higher scores are indicative of lower levels of the desired construct (for example, higher scores on the SF-36 indicate lower levels of disability) will be subtracted from the maximum value of that measure ( Higgins & Green, 2008). If no standard deviations are reported, they will be calculated from standard errors or confidence intervals that are available. Where suitable data are unavailable, study authors will be contacted in an attempt to retrieve data. If these data are not retrievable, such studies will not be included.

### Planned methods of analysis

This review will generate comparisons of psychotherapies delivered online and in person with a view to determining which psychotherapy most effectively reduces pain interference and psychological distress respectively in a chronic pain population.

***Exploratory analysis (Pair-wise meta-analyses).*** Where data are available, pairwise meta-analyses (using Stata 13) of each comparison will act as an exploratory analysis. In the event that significant heterogeneity is discovered, a random effects model will be used. Forest plots will be used to illustrate the individual and pooled effect sizes.

***Network meta-analysis.*** A random effects NMA will be generated on Stata 13. The model will take an exploratory frequentist approach, whereby the parameter estimates observed within each study are used to make inferences regarding the relative efficacy of each treatment without priori hypotheses. Pooled SMDs and 95% confidence intervals will respectively be used to quantify the magnitude of difference between each intervention type and the uncertainty surrounding these differences. Direct estimates will be generated based on interventions directly compared in trials, indirect estimates will be generated based on the results of direct estimates where intervention types that have not been directly compared share a common comparator, and network estimates will be generated through combining direct and indirect estimates ( Al Khalifah *et al.*, 2018). A network forest plot and interval plot will be created to graphically represent the effect size of each included study. Treatment rankings and SUCRA cumulative ranking probabilities will be created to identify the most effective psychotherapies

***Classification of arms***: For the primary analysis, studies will be grouped based on the type of psychotherapy employed and the modality of delivery (for example, in person vs. online CBT). Each category must have data from more than one study to warrant inclusion in the network. Otherwise, such studies will be categorised as “other” and further subcategorised based on the delivery modality. For studies employing combinations of psychotherapeutic techniques that do not coincide with a specific intervention format, such trials will be grouped in a “multimodal” category which will be further subcategorized based on the delivery modality. As low statistical power between comparisons is typically a concern when conducting NMAs ( Thorlund & Millis, 2012) and large numbers of interventions employing unconventional and unique psychotherapeutic formats are anticipated, the purpose of this categorisation procedure is to avoid low statistical power between trial comparisons. Studies utilising a medium of psychotherapy delivery that does not meet our inclusion criteria (such as over the phone or teleconferencing) will be categorised as “other delivery modalities”, providing that at least one arm in that study employs an eligible delivery modality. Trial arms including WLC or TAU will be categorised as inactive controls whereas arms assessing interventions without identifiable psychological components (for example, exercise, education and medical therapy) will be categorised as active controls.

### Statistical heterogeneity and inconsistency

Given the variety in study length, engagement required, and samples used, notable heterogeneity is anticipated. Statistical heterogeneity will be assessed using the *I* ^2^ statistic, which calculates the percentage of variability due to heterogeneity and not chance. Where substantial heterogeneity is evident between trials ( *I* ^2^>40%), subgroup analyses and meta-regressions will respectively be considered to determine the extent to which categorical and continuous covariates influenced the effect sizes generated, such as the type of chronic pain, length of morbidity, pain severity at baseline, and the duration of the intervention. Within the NMA, global incoherence (i.e., the extent to which the direct and indirect estimates are in agreement) will be assessed using the Wald statistic, and a local test will be conducted to investigate loop incoherence.

### Risk of bias across studies

As part of the exploratory analysis, funnel plots of both outcomes will be generated in Stata 13. These plots will be assessed for symmetry to determine the presence of publication bias. The Egger Test will also be conducted using Stata 13, to investigate whether study size is related to the study estimate.

### Additional analyses

The influence of studies at a high risk of bias will be investigated by removing them from the pairwise and network meta-analyses one at a time. Studies with at least one high risk of bias domain will be considered to be of high risk and removed from the analysis.

### Dissemination of information

We intend on disseminating the findings through traditional academic platforms including peer-reviewed journals and academic conferences. We will also disseminate the findings through the Centre for Pain Research’s public facing channels (such as our website and social media). In doing so, we hope to facilitate the dissemination of our findings to all stakeholders including clinicians, researchers, and chronic pain patients.

### Study status

We have finished the search and study screening phases and are currently preparing to extract data from the included studies. Following data extraction, studies will be categorised based on the treatment type and delivery modality. Following study categorization, the analyses, quality of evidence assessment, and write up will be conducted.

## Discussion

### Contribution to literature

It is generally accepted that psychotherapies are effective in assisting pain management for a chronic pain population. Furthermore, there has been an increase in the number and variety of psychotherapies delivered online for chronic pain. Although research has been done in this area ( Tang, 2018; Vugts *et al.*, 2018), to date, in the context of chronic pain, there is no clear indication as to which psychotherapy delivered either in person or online is the most effective. The proposed review will address this gap in the literature.

This review will extend previous research in the area by quantitatively comparing a variety of psychotherapies delivered both online and in person to identify the most efficacious in the context of chronic pain. Specifically, the NMA will return rankings, determining which psychotherapy and modality has been the most effective in reducing the primary outcome, pain interference. Such information will guide clinicians, as they choose psychotherapies to use when delivering an intervention for chronic pain.

As the treatment rankings yielded by the NMA will indicate the relative efficacy and cost-effectiveness of different psychological treatments for chronic pain management, the rankings will enable treatment providers to select the most suitable treatment options for their patients. For example, if a particular intervention is found to be very effective but expensive to implement, it would be important for clinicians to know the following most effective treatment, while keeping the cost-effectiveness of each intervention into consideration. In much the same way the rankings will be an aid to researchers choosing components for chronic pain studies. Additionally, through implementing the GRADE criteria, this review will provide an indication of the methodological quality of the available research in psychotherapy for chronic pain management, and the extent to which the methodological quality could have influenced the findings.

### Limitations

A significant degree of heterogeneity is anticipated due to trials being grouped, regardless of variations in intervention format (e.g. online, in-person, with/without therapist contact, number of sessions) or sample characteristics (e.g. age, socio-economic status, degree of pain interference, comorbidities). This could lead to high uncertainty surrounding the effect estimates and consequently bias any inferences ( Riley *et al*., 2017). Additionally, as studies will be grouped regardless of chronic pain-type, the generalizability of the findings towards specific diagnoses will be compromised. As all studies employing multiple psychotherapeutic techniques will be categorized as “multimodal” and all studies employing unconventional psychotherapeutic strategies will be categorised as “other”, this could result in further heterogeneity between the included trials. Additionally, deciphering the effective components of such categories will be impossible using this categorisation procedure. Although research examining the impact of language restrictions within meta-analyses is mixed ( Jüni *et al*., 2002; Wang *et al*., 2015), this inclusion criterion introduces the possibility of language bias into the review. As only follow-up data will be synthesised, the findings will provide no insight into the long-term sustainability of the treatments. As recipients of psychological interventions are unable to be blinded to their treatment condition, all of the studies incorporated in this review are susceptible to detection and allegiance biases ( Munder *et al*., 2011). Although measures will be taken to assess the magnitude of publication bias, only including peer-reviewed articles could increase publication bias. Additionally, as this review focuses solely on self-report measures, all included studies are susceptible to response bias or social desirability ( Rosenman *et al*., 2011). These inherent biases could consequently inflate the treatments’ effects ( Shean, 2014).

### Implications of the review

This review will, to our knowledge, provide the first comprehensive overview of the relative effectiveness of various psychotherapeutic interventions for chronic pain. This review will also provide a novel understanding of how the medium of psychotherapy delivery influences the effectiveness of such interventions. These insights can inform healthcare professionals on the most effective treatment route for improving health related quality of life amongst chronic pain patients. Moreover, as online psychotherapeutic interventions have been found to be a cost-effective modality of treatment delivery ( Lenhard *et al.*, 2017) and financial constraints are typically reported barriers to accessing non-pharmacological treatment in chronic pain sufferers ( Becker *et al.*, 2017), any findings indicating preferable effectiveness of online treatment could encourage increased treatment using this modality and reduce financial burdens to society and chronic pain patients.

## Data availability

### Underlying data

No data are associated with this article

### Extended data

Open Science Framework: Chronic pain psychotherapies delivered online and in person: systematic review and network meta-analysis. https://doi.org/10.17605/OSF.IO/Q9NGC ( O’Connor, 2019)

This project contains the following extended data:

- NMA 2 Additional File 1.docx (Study search strategy)

### Reporting guidelines

PRISMA-P checklist ‘An analysis of psychotherapies delivered online and in person for patients with chronic pain: protocol for a systematic review and network meta-analysis’. https://doi.org/10.17605/OSF.IO/Q9NGC ( O’Connor, 2019)

Data are available under the terms of the Creative Commons Zero "No rights reserved" data waiver (CC0 1.0 Public domain dedication).
