# Supplementary material for: The relative effectiveness of psychotherapeutic techniques and delivery modalities for chronic pain: a protocol for a systematic review and network meta-analysis
Source: HRB Open Res. 2020 Aug 17;2:25. Originally published 2019 Oct 24. [Version 2] doi: 10.12688/hrbopenres.12953.2 (PMC7459872; doi:10.12688/hrbopenres.12953.2)
Supplement: Peer review history

# Peer review: Reviewer response for version 2

**DOI:** 10.21956/hrbopenres.14243.r27826

**Published:** 2020-08-27

**Authors:** Gordon H. Guyatt, Long Ge

**Affiliations:** 1 School of Public Health, Lanzhou University, Lanzhou, China; 2 Department of Medicine, McMaster University, Hamilton, Canada

We have no further comments to make.

Is the study design appropriate for the research question?

Yes

Is the rationale for, and objectives of, the study clearly described?

Partly

Are sufficient details of the methods provided to allow replication by others?

Partly

Are the datasets clearly presented in a useable and accessible format?

Not applicable

Reviewer Expertise:

Evidence-based medicine.

We confirm that we have read this submission and believe that we have an appropriate level of expertise to confirm that it is of an acceptable scientific standard.

# Peer review: Reviewer response for version 1

**DOI:** 10.21956/hrbopenres.14035.r27058

**Published:** 2020-01-30

**Authors:** Gordon H. Guyatt, Long Ge

**Affiliations:** 1 School of Public Health, Lanzhou University, Lanzhou, China; 2 Department of Medicine, McMaster University, Hamilton, Canada

This review is a protocol for a systematic review and network meta-analysis aiming to evaluate psychotherapies delivered in person and online for chronic pain patients. This protocol is well-written and the reporting quality is good. However, the objective and methods need further improvement. Our suggestions follow.

1. In the first view of the title, it appears the study aims to compare online and in person methods of psychotherapy, mainly focusing on the differences between delivered in person and online. However, in the methods section, we find that authors also compare different types of psychotherapies. Therefore, the authors should make a clearer study objective and more suitable title.
2. The authors plan to use GRADE to assess the certainty (quality) of evidence. However, they do not refer to the latest advance of GRADE for NMA. We suggest that authors should carefully read the recent GRADE papers and make substantial revisions accordingly. In particular, they should plan to consider what they will do if they find sparse networks and the assumption of common heterogeneity leads to counterintuitive blowing up of the network estimate confidence intervals relative to those from the direct comparisons. Some references follow ^1^^-^^3^.
3. The authors should provide more detailed statistical analyses. First, how do the authors obtain direct, indirect, and NMA estimates? Second, how do the authors assess the transitivity? Thirdly, inconsistency assessment needs more details (if using GRADE they should probably use GRADE terminology, and use “incoherence” when they refer to differences between direct and indirect estimates). Finally, do they have any hypotheses regarding possible explanations of heterogeneity and if so what is the postulated direction of effect and are there any planned subgroup analysis or meta-regression to explore the source of heterogeneity?

Minor suggestions:

1. Although the authors will search the clinical trial registers, they only include studies published in peer-review journals. They should consider the possibility of publication bias.
2. The authors plan to exclude any interventions that don’t directly connected to the network. However, this is a systematic review and NMA, they should include such interventions and, in addition to the NMA, summarize results.
3. The words used should remain consistent throughout the paper. For example, the authors use “quality” to describe the risk of bias of individual RCT in abstract, but “risk of bias” is used in method section. They should stick to risk of bias for individual studies, and restrict quality to the overall body of evidence.
4. The description on benefit and risk of NMA is not about the methods; they should delete or move to discussion section.
5. If SD is still unavailable after attempting to all methods mentioned, what do the authors plan to do? Do they plan to use another SD imputation method?
6. Global test of inconsistency should be calculated.
7. Sensitivity analysis excluding studies with high risk of bias will be planned only for pairwise meta-analysis, but it also should be performed for NMA. In addition, how do the authors classify individual studies into high and low risk of bias?
8. There are now two Cochrane risk of bias instruments for RCTs. The authors propose to use the older one. It has advantages over the newer one but it has an important disadvantage: the unclear risk of bias categorization is problematic. It turns out that people can make reasonable inferences regarding risk of bias when it is unclear (see Akl *et. al*. 2012 ^4^). The authors should consider using a revised Cochrane risk of bias instrument (see Guyatt GH, Busse JW. Modification of Cochrane Tool to assess risk of bias in randomized trials)
9. If reviewers disagree on risk of bias, how will authors deal with the disagreement?
10. The authors propose to screen 10% of the articles in duplicate. Is this for title and abstracts, for full text, or both? They propose to do this to “to ensure consistency”. For both titles and abstracts and full text,, how many do they expect 10% will represent. How did they choose 10% - what is the justification? What is their standard for satisfactory consistency? If they do not achieve this standard what is their plan?

Is the study design appropriate for the research question?

Yes

Is the rationale for, and objectives of, the study clearly described?

Partly

Are sufficient details of the methods provided to allow replication by others?

Partly

Are the datasets clearly presented in a useable and accessible format?

Not applicable

Reviewer Expertise:

Evidence-based medicine.

We confirm that we have read this submission and believe that we have an appropriate level of expertise to confirm that it is of an acceptable scientific standard, however we have significant reservations, as outlined above.

## References

[1] : Advances in the GRADE approach to rate the certainty in estimates from a network meta-analysis. *J Clin Epidemiol*.93: 10.1016/j.jclinepi.2017.10.005 36-44 10.1016/j.jclinepi.2017.10.005 29051107

[2] : GRADE approach to rate the certainty from a network meta-analysis: avoiding spurious judgments of imprecision in sparse networks. *J Clin Epidemiol*.105: 10.1016/j.jclinepi.2018.08.022 60-67 10.1016/j.jclinepi.2018.08.022 30253217

[3] : GRADE approach to rate the certainty from a network meta-analysis: addressing incoherence. *J Clin Epidemiol*.2019;108: 10.1016/j.jclinepi.2018.11.025 77-85 10.1016/j.jclinepi.2018.11.025 30529648

[4] : Specific instructions for estimating unclearly reported blinding status in randomized trials were reliable and valid. *J Clin Epidemiol*.2012;65(3) : 10.1016/j.jclinepi.2011.04.015 262-7 10.1016/j.jclinepi.2011.04.015 22200346

# Response

**Published:** 2020-08-10

**Authors:** Laura O'Connor

**Affiliations:** National University of Ireland, Galway, Ireland

Drs Ge and Guyatt,

 Thank you for this review of our protocol, we appreciate the time you have taken and your detailed feedback. Below, we respond to your specific points.

- In the first view of the title, it appears the study aims to compare online and in person methods of psychotherapy, mainly focusing on the differences between delivered in person and online. However, in the methods section, we find that authors also compare different types of psychotherapies. Therefore, the authors should make a clearer study objective and more suitable title.

We have edited the title to highlight that the review intends to compare different psychotherapeutic techniques with one another, in addition to the modality by which these techniques are delivered.

  

- The authors plan to use GRADE to assess the certainty (quality) of evidence. However, they do not refer to the latest advance of GRADE for NMA. We suggest that authors should carefully read the recent GRADE papers and make substantial revisions accordingly. In particular, they should plan to consider what they will do if they find sparse networks and the assumption of common heterogeneity leads to counterintuitive blowing up of the network estimate confidence intervals relative to those from the direct comparisons. Some references follow 1- 3.

We have edited this section to include the latest advancements in the GRADE criteria for NMAs based on the citations provided.

  

- The authors should provide more detailed statistical analyses. First, how do the authors obtain direct, indirect, and NMA estimates? Second, how do the authors assess the transitivity? Thirdly, inconsistency assessment needs more details (if using GRADE they should probably use GRADE terminology, and use “incoherence” when they refer to differences between direct and indirect estimates). Finally, do they have any hypotheses regarding possible explanations of heterogeneity and if so what is the postulated direction of effect and are there any planned subgroup analysis or meta-regression to explore the source of heterogeneity?

We have added more detail into the analysis section describing how the direct, indirect and network estimates will be generated. We have also edited the language regarding the GRADE terminology. We have highlighted that we anticipate heterogeneity and made clear that subgroup analyses and meta-regressions will be considered in the presence of substantial heterogeneity. These analyses will also determine whether the assumption of transitivity will be met.

 ​​​​​​​

 Minor suggestions:

1. Although the authors will search the clinical trial registers, they only include studies published in peer-review journals. They should consider the possibility of publication bias.
2. The authors plan to exclude any interventions that don’t directly connected to the network. However, this is a systematic review and NMA, they should include such interventions and, in addition to the NMA, summarize results.
3. The words used should remain consistent throughout the paper. For example, the authors use “quality” to describe the risk of bias of individual RCT in abstract, but “risk of bias” is used in method section. They should stick to risk of bias for individual studies, and restrict quality to the overall body of evidence.
4. The description on benefit and risk of NMA is not about the methods; they should delete or move to discussion section.
5. If SD is still unavailable after attempting to all methods mentioned, what do the authors plan to do? Do they plan to use another SD imputation method?
6. Global test of inconsistency should be calculated.
7. Sensitivity analysis excluding studies with high risk of bias will be planned only for pairwise meta-analysis, but it also should be performed for NMA. In addition, how do the authors classify individual studies into high and low risk of bias?
8. There are now two Cochrane risk of bias instruments for RCTs. The authors propose to use the older one. It has advantages over the newer one but it has an important disadvantage: the unclear risk of bias categorization is problematic. It turns out that people can make reasonable inferences regarding risk of bias when it is unclear (see Akl *et. al*. 2012 4). The authors should consider using a revised Cochrane risk of bias instrument (see Guyatt GH, Busse JW. Modification of Cochrane Tool to assess risk of bias in randomized trials)
9. If reviewers disagree on risk of bias, how will authors deal with the disagreement?
10. The authors propose to screen 10% of the articles in duplicate. Is this for title and abstracts, for full text, or both? They propose to do this to “to ensure consistency”. For both titles and abstracts and full text,, how many do they expect 10% will represent. How did they choose 10% - what is the justification? What is their standard for satisfactory consistency? If they do not achieve this standard what is their plan?

 

 We have edited the document based on the majority of the minor edits suggested. We have highlighted the potential for publication bias within the limitations section, removed the section outlining the benefits of NMA from the methods section, and included how we will calculate global inconsistency. We have also made clear that studies without dispersion values to facilitate an SD imputation will be excluded, how disagreements regarding the risk of bias will be resolved, and how studies will be categorised as being high risk of bias.

 We have explained that only abstract screening will be done in duplicate. We do not have a justification for 10% of studies being screened in duplicate. As a large number of abstracts are anticipated, this proportion was decided based on the resources available.

 We intend on using the older version of the Cochrane Risk of Bias as the researchers are not familiar with inferring risk of biases without the relevant information being reported in trials. The unclear risk of biases will be taken into consideration when judging the trustworthiness of each study’s results.

 Thank you again for your thoughtful feedback.

# Peer review: Reviewer response for version 1

**DOI:** 10.21956/hrbopenres.14035.r26884

**Published:** 2019-11-18

**Authors:** Miel A. P. Vugts

**Affiliations:** 1 Tranzo Scientific Center for Care and Welfare, Tilburg School of Social and Behavioral Sciences, Tilburg University, Tilburg, The Netherlands

The authors describe a network meta-analysis for determining which psychotherapy (approach) and mode of delivery (internet-based versus in-person) is most efficacious for patients with chronic pain. The question of how psychotherapy forms can be ranked in terms of efficacy is both relevant and complex. The protocol is clearly written down and PRISMA guidelines for systematic reviews and the extension for network meta-analyzes are followed. Limitations are well discussed.

 In my opinion, the article can be improved on several points.

- The writing style makes it easy to read. However, vague language is sometimes used. For example, "studies will be managed in Endnote".
- The paragraphs about evidence for the efficacy of (online) psychotherapy largely focusses on statistical significance of previous studies. Maybe, a more nuanced description can be given, also mentioning the effect sizes and methodological quality of previous research and its implications: how about potential risks of bias assessed in previous reviews, and clinical relevance of psychotherapies for chronic pain (in general)?
- Another potential area for improvement is the specific rationale for conducting a NMA: The statement "There has also been an increase in internet delivered interventions for chronic pain, however, there is a dearth of research evaluating the efficacy of online psychotherapies in comparison to their counterparts delivered in person. The current review will investigate these comparisons using a meta-analysis (NMA)" could also be read as a plea for more primary research on direct comparisons of psychotherapies via internet or in-person.
- The objective has been formulated in terms of a method, comparing treatments is a means and not an end in itself. I suggest to formulate the objective in terms of a theoretical problem: what exactly does one want to learn?
  Thus, the research question is not explicitly stated. The aim, as stated in the abstract ('evaluate and rank psychotherapies') is very ambitious. To this end, better primary evaluation studies might be needed more. Bluntly said, there is still a considerable risk of 'garbage in, garbage out'. Nonetheless, I agree that this NMA can provide complementary insights to the literature.

Suggestions are:

1. Explain a bit more about variation through 'intervention format or sample characteristics'. For example, patients with depression are sometimes included and sometimes not, the recruitment base can be 'open' (from the general public via internet) or 'closed' (from clinics or work settings), patients with particularly high education levels and computer self-efficacy to self-select for internet-delivered formats. Fidelity of implementation (not only attrition) can be an influence.
  Is it possible to take account of some moderating factors in the NMA? Will there be enough information per type of psychotherapy to do so?
2. Formulate clearer (theory-based) research questions and hypotheses (can you specify the "hypothesized values of the parameters"?). Since there is so much uncertainty about the processes by which psychotherapies are effective, I consider it to be legitimate to use NMA more as an exploratorative tool.  
3. More details could be added with respect to:
  - Inclusion and exclusion criteria:
  I suggest that you be more precise: Report clear criteria or exhaustive lists instead of examples of "psychotherapeutic content" and "debilitating diseases". This leaves questions like: is expressive writing instruction psychotherapeutic content? Is arthritis a debilitating disease?
  The choice of another study to exclude patients with headache is not an actual argument to do the same.
  Maybe, add a paragraph with specific a priori classifications of how, and on what basis, different kinds of psychotherapy are based on approaches, techniques, and delivery modes.
  Are there any criteria with regard to the methods that studies have used to screen the patients?
  - Which systematic reviews will be searched to identify additional relevant studies?
4. Describe how you will deal with risk of bias and small sample sizes of included studies in the NMA.

Minor points:

- In "this review will use PICO to frame and report review criteria:", the second "review" could be replaced by "eligibility".
- Add a reference to support: "However, when appropriately and conservatively used to synthesise research and aid decision making, they are extremely beneficial and influential".
- "Cost-effectiveness": Is this really within the scope of this article?

To note, I have experience with conducting meta-analyses, but I have no expertise in NMA specifically.

Is the study design appropriate for the research question?

Partly

Is the rationale for, and objectives of, the study clearly described?

Partly

Are sufficient details of the methods provided to allow replication by others?

Partly

Are the datasets clearly presented in a useable and accessible format?

Not applicable

Reviewer Expertise:

eHealth, chronic pain.

I confirm that I have read this submission and believe that I have an appropriate level of expertise to confirm that it is of an acceptable scientific standard.

# Response

**Published:** 2020-08-10

**Authors:** Laura O'Connor

**Affiliations:** National University of Ireland, Galway, Ireland

Dr Vugts,

 Thank you for taking the time to review our protocol, and for your very helpful feedback. We have followed your guidance and made several changes to the article, which we agree strengthens it. Below, we address your points in turn and indicate which specific changes we have made.

*The writing style makes it easy to read. However, vague language is sometimes used. For example, "studies will be managed in Endnote".*

 We have edited the text to use clearer language and better explain specifics.

*The paragraphs about evidence for the efficacy of (online) psychotherapy largely focusses on statistical significance of previous studies. Maybe, a more nuanced description can be given, also mentioning the effect sizes and methodological quality of previous research and its implications: how about potential risks of bias assessed in previous reviews, and clinical relevance of psychotherapies for chronic pain (in general)?*

*Another potential area for improvement is the specific rationale for conducting a NMA: The statement "There has also been an increase in internet delivered interventions for chronic pain, however, there is a dearth of research evaluating the efficacy of online psychotherapies in comparison to their counterparts delivered in person. The current review will investigate these comparisons using a meta-analysis (NMA)" could also be read as a plea for more primary research on direct comparisons of psychotherapies via internet or in-person.*

 We have edited this section to state our rationale for carrying out an NMA more clearly, and separately from the need for more primary research on this topic.

*The objective has been formulated in terms of a method, comparing treatments is a means and not an end in itself. I suggest to formulate the objective in terms of a theoretical problem: what exactly does one want to learn?*

*Thus, the research question is not explicitly stated. The aim, as stated in the abstract ('evaluate and rank psychotherapies') is very ambitious. To this end, better primary evaluation studies might be needed more. Bluntly said, there is still a considerable risk of 'garbage in, garbage out'. Nonetheless, I agree that this NMA can provide complementary insights to the literature.*

 We have reformulated the objective to more explicitly state what the question that this NMA aims to answer. We intend to be clear about the need for more high-quality primary research as well as the need for syntheses like this in reporting our results, as we expect that findings based on indirect evidence will need to be supported by direct research before strong conclusions can be drawn.

 ​​​​​​​

 Suggestions are:

1. *Explain a bit more about variation through 'intervention format or sample characteristics'. For example, patients with depression are sometimes included and sometimes not, the recruitment base can be 'open' (from the general public via internet) or 'closed' (from clinics or work settings), patients with particularly high education levels and computer self-efficacy to self-select for internet-delivered formats. Fidelity of implementation (not only attrition) can be an influence.Is it possible to take account of some moderating factors in the NMA? Will there be enough information per type of psychotherapy to do so?*We have added examples to better explain these variations. In terms of moderating factors, subgroup analyses and meta-regressions will be considered should substantial heterogeneity be evident.
2. *Formulate clearer (theory-based) research questions and hypotheses (can you specify the "hypothesized values of the parameters"?). Since there is so much uncertainty about the processes by which psychotherapies are effective, I consider it to be legitimate to use NMA more as an exploratorative tool.  *We have edited that section to highlight that the NMA is an exploratory analysis, where we are using the observed parameter estimates of the various treatments to generate comparisons without any priori hypotheses.
3. More details could be added with respect to: *- Inclusion and exclusion criteria:I suggest that you be more precise: Report clear criteria or exhaustive lists instead of examples of "psychotherapeutic content" and "debilitating diseases". This leaves questions like: is expressive writing instruction psychotherapeutic content? Is arthritis a debilitating disease?*We have edited our language and added examples or additional explanation where needed *The choice of another study to exclude patients with headache is not an actual argument to do the same.*We have amended this to better explain our rationale. *Maybe, add a paragraph with specific a priori classifications of how, and on what basis, different kinds of psychotherapy are based on approaches, techniques, and delivery modes.*Amended *Are there any criteria with regard to the methods that studies have used to screen the patients?*We are constrained by the literature on this - it is likely that individual papers will describe their methods screening of participants in varying levels of detail, and with little consistency across papers as a whole. In many cases only screening criteria are covered, not the methods used to apply those criteria. *Which systematic reviews will be searched to identify additional relevant studies?*Should systematic reviews be identified by the search that are eligible for inclusion, their references will be searched.
4. *Describe how you will deal with risk of bias and small sample sizes of included studies in the NMA.*Our risk of bias and quality of evidence sections detail how we will deal with these elements.

Minor points:

- *In "this review will use PICO to frame and report review criteria:", the second "review" could be replaced by "eligibility".*Edited
- *Add a reference to support: "However, when appropriately and conservatively used to synthesise research and aid decision making, they are extremely beneficial and influential"*This section has since been revised, removing this sentence.
- *"Cost-effectiveness": Is this really within the scope of this article?*This is a fair point - a full cost-effectiveness comparison is not within the scope of this project, not least because the necessary information is unlikely to be reported within the literature. However, we are acknowledging that any comparison will allow for a decision to be made taking cost into consideration - for example our results may rank something highly that is prohibitively costly for a particular setting, but can offer the next ranked treatments as possible alternatives, making cost a factor but not a focus.

Thank you again for your time and for the useful feedback you have given us.
